# A radiographic analysis of humerus shaft fractures to predict non-union

**DOI:** 10.1007/s11845-025-03905-8

**Published:** 2025-02-15

**Authors:** Alexander Price, Conor O’Driscoll, Nicolaas Kotze, Danilo Vukanic, Petr Jemelik, May Cleary, David O’Briain

**Affiliations:** 1 Department of Trauma and Orthopaedic Surgery, University Hospital Waterford, Waterford, X91ER8E Ireland; 2 Royal College of Surgeons in Ireland, Dublin, Ireland

**Keywords:** Conservative treatment, Displacement, Fractures, Non-union, Surgery

## Abstract

**Background:**

Humeral shaft fracture non-union rates of up to 33% have been reported when managed non-operatively. When managed surgically, non-union rates of 10% have been seen. The initial radiographic displacement parameters may be of significance in determining whether non-operative treatment might fail.

**Aims:**

To determine whether the initial radiographic displacement of humeral shaft fractures can predict non-union rates and assist in decision-making for surgical intervention.

**Methods:**

A retrospective cohort study was performed to identify all patients with humeral shaft fractures presenting to a regional trauma centre over a three-year period. Three-observer measurements were taken on defined radiographic parameters. These were compared between groups treated successfully with surgery and with non-operative intervention. A subset group was identified who failed non-operative treatment and required delayed surgery. Statistical analysis was performed to determine whether the group that failed non-operative treatment met the proposed radiographic parameters predicting treatment failure.

**Results:**

Eighty patients were identified over the defined three-year period. Failure of non-operative management occurred in 6/43 (13.95%) patients. Failed conservative treatment was associated with increased age, female gender, and increased AP translation of lateral radiographs. Fractures successfully treated non-operatively showed a significantly lower AP translation on the lateral radiographs compared to patients who failed non-operative treatment 9.69 mm (IQR 4.90–14.05 mm) versus 22.61 mm (IQR 15.73–23.83 mm), *p*-value = 0.042.

**Conclusion:**

Significant initial AP displacement may be associated with failure of non-operative management. This study highlights the importance of initial radiographic parameters of displacement in predicting possible failure of non-operative management for midshaft humerus fractures.

## Background

Humeral shaft fractures represent approximately 1–5% of all fractures and have an annual incidence of 13–20 per 100,000 people [1]. A bimodal distribution has been described with humeral shaft fractures, firstly occurring in younger males, most commonly secondary to high-energy trauma. Secondly, occurring in elderly females (ages 60–80) typically from low-energy falls [2]. Humeral shaft fractures are defined as fractures occurring between the surgical neck of the humerus and proximal to the epicondyles of the humerus [3]. Non-operative management of humeral shaft fractures with bracing became the established standard of care in the 1970’s and is still common practise [4]. Surgical management is more frequently utilised in modern practise, in particular for polytrauma patients, open fractures, vascular compromise, ipsilateral articular injuries, and fractures that fail non-operative management [5].

A recent systematic review of humeral shaft fracture management by Sargeant et al. demonstrated a significantly increased risk of non-union when non-operative treatment was undertaken compared to surgically treated fractures, with non-union occurring in 17.6% of patients managed non-operatively [6]. Recent studies have demonstrated that humeral shaft fractures managed non-operatively may have non-union rates of up to 33% [7]. Surgery is not without risk. Even with surgical intervention, non-union rates of 6.3% were noted, with some studies showing non-union rates as high as 10% [6, 7]. Despite this, surgical treatment has a significantly higher union rate [5–7]. To date, most studies assessing the failure of non-operative management of humeral shaft fractures have focused on patient factors and fracture morphology [7–9]. Kim et al. proposed that radiographic parameters may be associated with the success or failure of non-operative management [10]. They noted that failure of non-operative treatment was found to be associated with greater initial anterior–posterior (AP) angulation and medial/lateral (M/L) translation [10]. Our aim was to examine their hypothesis regarding the influence of initial displacement, which has yet to have been tested in other studies, against our patient population in a regional trauma unit in Ireland.

## Methods

A retrospective cohort study was performed to identify all patients with humeral shaft fractures (AO Classification: 12) who were treated at a regional trauma centre in the Republic of Ireland. All referrals made to the Orthopaedic Department are made using an electronic platform, which was used to identify and collate demographic information for patients included in the study. All patients managed operatively and non-operatively were identified between January 2021 and December 2023. Patients were either managed non-operatively with humeral bracing (Universal Humeral Brace, Beagle Orthopaedic, Lancashire, UK) or with primary surgical fixation, by either performing open reduction and internal fixation with plate and screw constructs (Large fragment LC-DCP plates, Synthes, PA, USA) or intramedullary nailing (Humeral Nailing System, Stryker, AZ, USA). Data were collected using the local electronic referral system, with specific keywords to identify all humeral shaft fractures over this time. Exclusion criteria included periprosthetic fractures, paediatric fractures (under 18), intra-articular fractures, and patients without serial imaging confirming fracture union.

Imaging was reviewed using the National Integrated Medical Imaging System (NIMIS), which is a system that.

allows for the viewing of patient radiographic investigations across the majority of hospitals providing orthopaedic trauma care nationwide, reducing the potential loss to follow up [11]. All patients that were included in this study had their imaging accessible within the institution the study was performed. Patient information was used from the electronic referral platform to view radiographic imaging to analyse the radiographic parameters of initial fracture displacement for all patients meeting the inclusion criteria. There was a total of 160 patients identified. Patients were excluded if they had any intra-articular extension, inadequate or incomplete radiographs, incorrectly coded non-humeral shaft fractures, the paediatric population, periprosthetic fractures, pathological fractures, any other ipsilateral upper limb fracture, or duplicate referrals. Adequacy of radiographs was determined by visualisation of the entire humerus in one radiograph with inclusion of the shoulder and elbow joints.

**Fig. 1:**
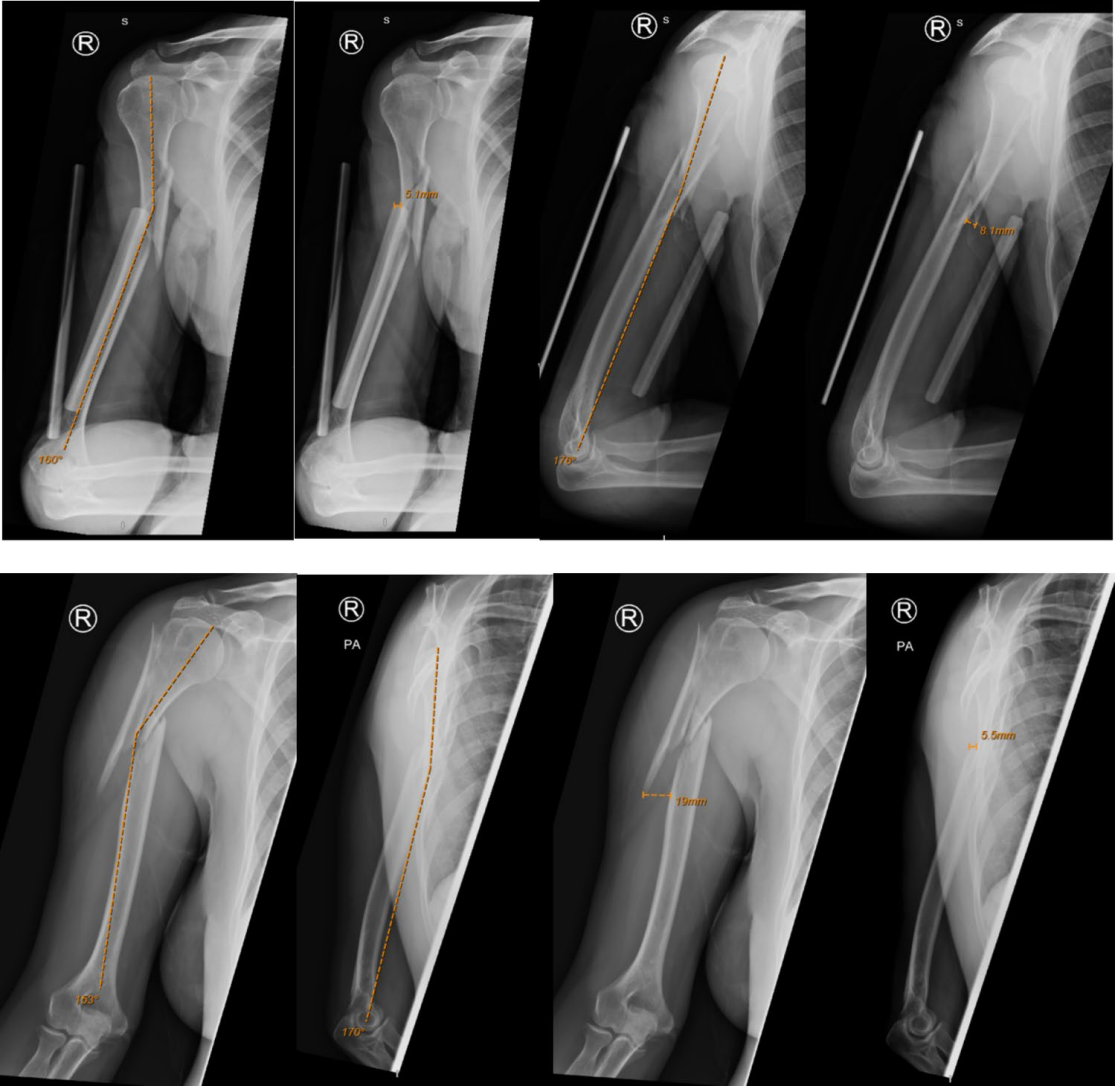
Displacement value analysis

Radiographic evaluation was performed by viewing an AP radiograph of the humerus and a lateral radiograph of the humerus. The AP radiograph was used to measure the M/L translation and the M/L angulation, and the lateral radiograph was used to measure the AP translation and AP angulation. The AP radiograph was also used to determine the angulation direction either being varus or valgus/neutral. The lateral radiograph was used to determine the apex direction of the fracture, either being anterior or posterior. It was important to note that these radiographs needed to include the shoulder and elbow joint within the same radiograph. The NIMIS systems inbuilt digital image analysis software was used to determine the displacement values (McKesson Enterprise Medical Imaging, Irving, TX, USA) (Fig. 1). Imaging was reviewed by three independent observers to ensure inter-observer reliability. Where differences in measurements were identified, the mean of the measurements was used for analysis.

Radiographic parameters taken included fracture location (proximal, middle, or distal shaft), the fracture pattern, medial/lateral angulation on AP view, angulation apex direction, medial/lateral translation on AP view, AP angulation on the lateral view, apex angulation on the lateral view, AP translation on lateral view, whether patients received primary surgical fixation or delayed surgical fixation, and the method of surgical fixation (Table 1).

**Table 1:** Radiographic parameters

Radiographic parameters assessed						
Fracture location	Proximal		Middle		Distal	
Fracture pattern	Transverse	Oblique	Spiral	Butterfly	Comminuted	Segmental
AP radiograph	M/L translation			M/L angulation		
Lateral radiograph	AP angulation			AP translation		
Apex angulation	Anterior			Posterior		
Method of surgical fixation	Open reduction internal fixation (ORIF)			Intramedullary nailing (IMN)		

The primary outcome of the study was the failure of non-operative management. Associated factors were recorded, such as fracture displacement and delayed union and non-union. Primary surgical fixation was defined as surgical intervention within four weeks (28 days) of the initial injury, where delayed surgery was at any time longer than four weeks. Non-union is defined as absent clinical or radiographic healing 9 months post injury without signs of progressive healing on radiographs three months apart [7, 10]. Delayed union was defined as failure to reach union by six months after the initial injury [7, 10]. Where delayed surgical fixation was decided upon, this decision was based on radiographic signs of delayed or non-union, in conjunction with clinical symptoms of delayed or non-union.

## Results

**Fig. 2:**
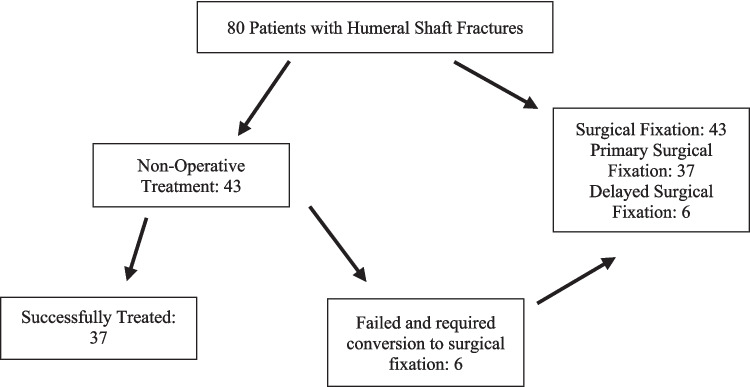
Flow diagram of treatment pathway

Eighty patients with humeral shaft fractures were identified over the study period. Then, 43 (53.75%) of the patients underwent surgical fixation. Failed non-operative treatment and conversion to delayed surgical fixation accounted for 6 (13.95%) of the 43 surgically managed patients. There were 37 (46.25%) patients who were successfully treated with non-operative management (Fig. 2.) The median age for the cohort of patients was 56.40 years (IQR 41.46–71.76) and the average age of failed conservative treatment was 60.61 years of age. Then, 34 (42.50%) of the patients were males and 4/6 of those who failed conservative treatment were female gender (Table 2).

**Table 2:** Demographic details

	**Primary surgery**	**Non-operative**	**Conversion**
Age	46.93 (40.91-52.96)	62.16 (56.02-68.83)	60.61 (44.13-77.08)
Gender (male/female)	19/18	13/24	2/4
Days waiting (days)	5.95 (1–24)	N/A	130 (65–309)

One patient who underwent primary surgical fixation was complicated by a periprosthetic fracture. This unfortunately required revision fixation. The fracture healed satisfactorily, and the patient suffered no further complications. When primary surgical fixation was undertaken, there was an average of 5.95 days between injury and surgical fixation. Where conversion from non-operative treatment to surgical fixation was decided upon, there was an average time between injury and fracture fixation of 130 days (range 65 to 309 days).

### Fracture characteristics

When evaluating the fracture location, it was noted that 43 (53.1%) affected the middle third, 24 (29.6%) the proximal third, and 14 (17.3%) were distal third fractures. This was comparable to current literature where Tytherleigh-Strong et al. noted that the middle third of the diaphysis fractures occurred in 60% of cases, followed by 30% in the proximal third and the remaining 10% in the distal third [2].

**Fig. 3:**
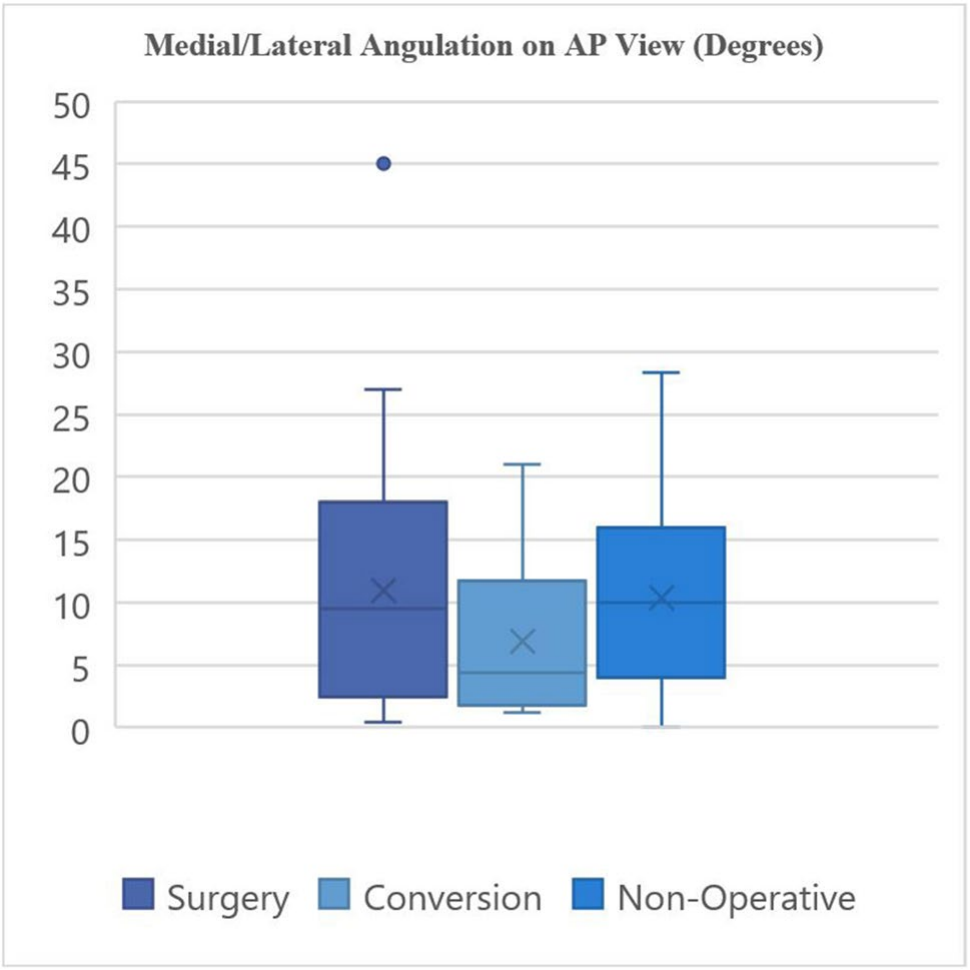
Radiographic characteristics

**Fig. 4:**
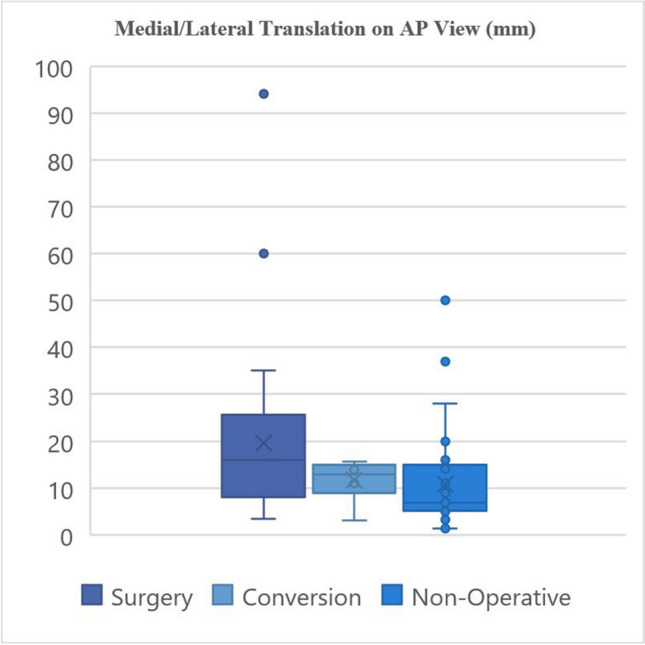
Radiographic characteristics

**Fig. 5:**
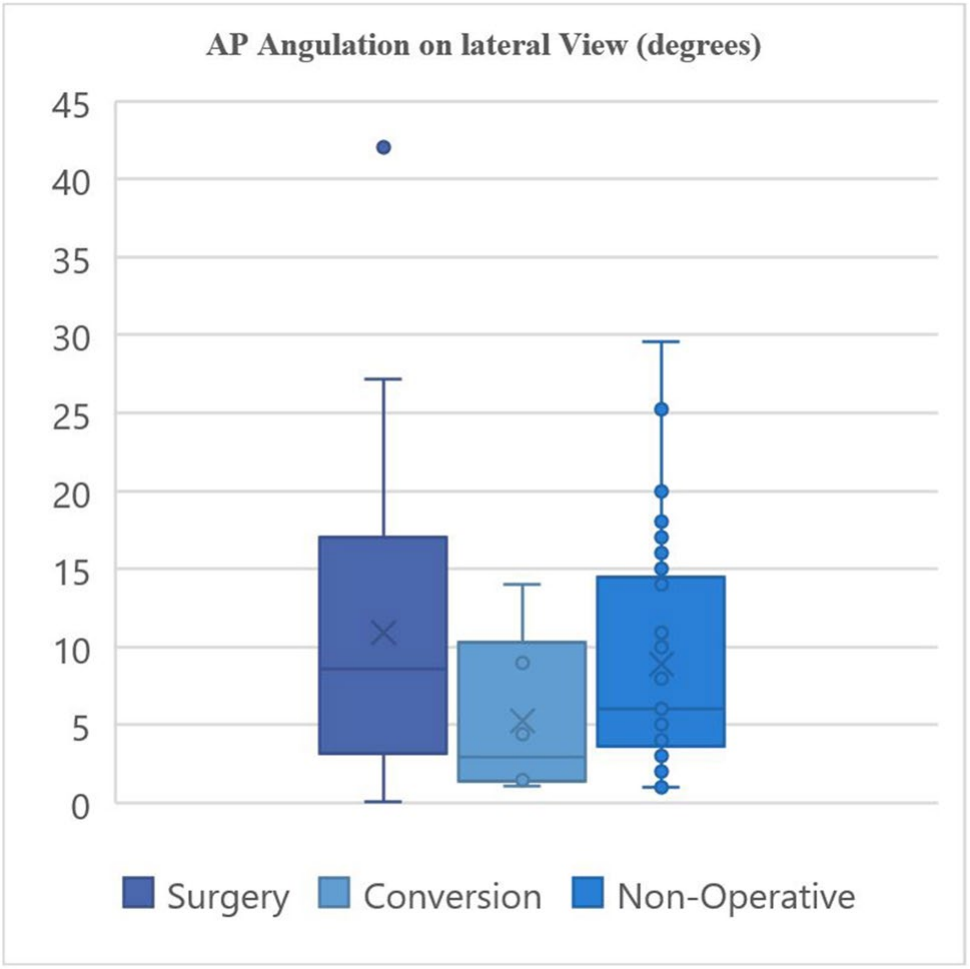
Radiographic characteristics

**Fig. 6:**
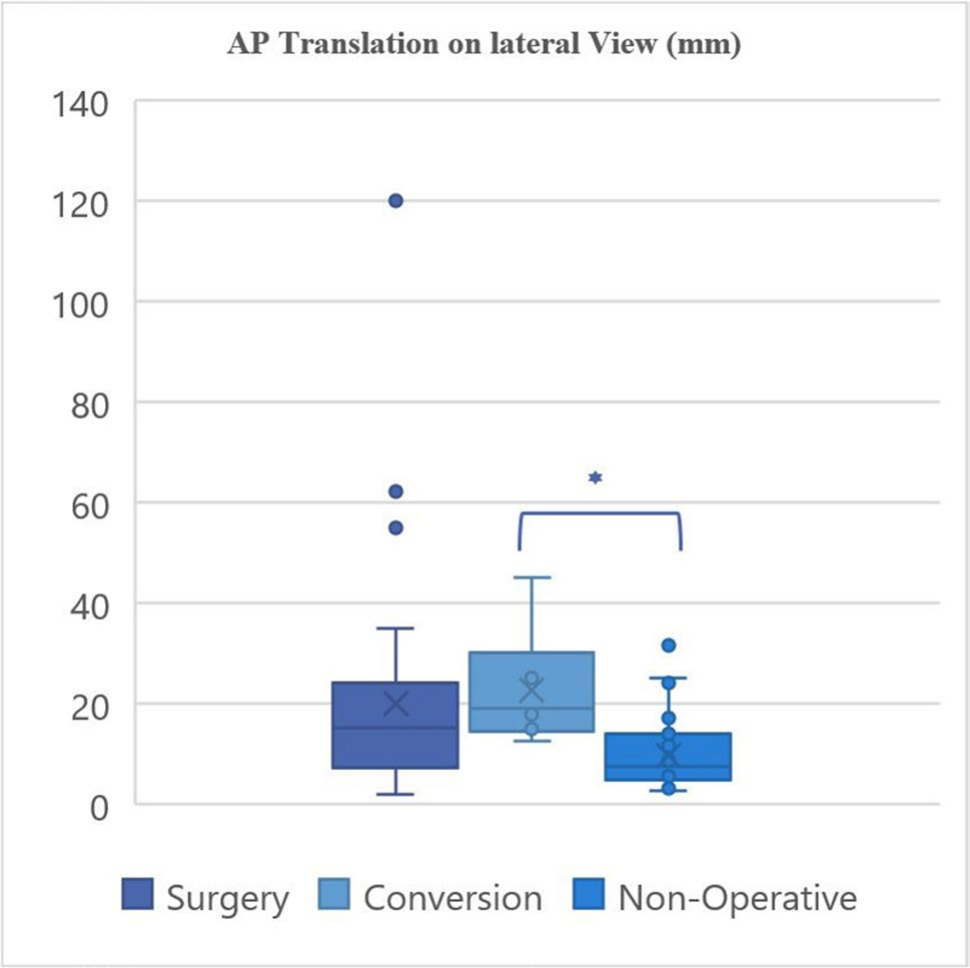
Radiographic characteristics

The radiographic characteristics of the groups are provided in Tables 3 and 4 and demonstrated graphically in Fig. 3, 4, 5, and 6.

**Table 3:** Fracture characteristics

	**Surgery**	**Non-operative**	**Conversion**
**Fracture location:**			
Proximal	9	13	1
Middle	15	23	5
Distal	13	1	0
**Fracture characteristics:**			
Transverse	5	4	3
Oblique	3	7	0
Spiral	13	14	1
Butterfly	14	9	2
Comminuted	1	2	0
Segmental	1	1	0
**Apex angulation:**			
Anterior	22	28	4
Posterior	15	9	2
**Angulation direction:**			
Valgus/neutral	10	14	2
Varus	27	23	4

**Table 4:** Fracture displacement values

	**Medial/lateral angulation on AP view (degrees) **	**Medial/lateral translation on AP view (mm) **	**AP angulation on lateral view (degrees) **	**AP translation on lateral view (mm) **
Primary surgical fixation	10.97°, 9.47°	19.67 mm, 16.0 mm	11.53°, 8.62°	20.02 mm, 15.2 mm
	(2.5–18°)	(9.4–25.4 mm)	(3.38–17.00°)	(7.40–22.9 mm)
	1–45°	3.2–94 mm	0.1–23°	1.8–55 mm
Non-operative management	10.37°, 10.00°	10.89 mm, 6.80 mm	8.87°, 6.00°	9.69 mm, 7.50 mm
	(4–16°)	(5.10–15.0 mm)	(3.65–14.5°)	(4.90–14.05 mm)
	0–28.3°	1.4–50 mm	1.0–29.6°	1.5–31.5 mm
Conversion	6.94°, 4.4°	11.73 mm, 12.95 mm	5.25°, 2.95°	22.61 mm, 19.10 mm
	(2.45–7.73°)	(11.25–14.58 mm)	(1.48–7.85°)	(15.73–23.83 mm)
	1.2–8.64°	3.0–15.7 mm	1.1–14.0°	12.5–45 mm
*p*-value	0.329	0.740	0.172	0.042

*Mean, median; IQR; range

**Fig. 7:**
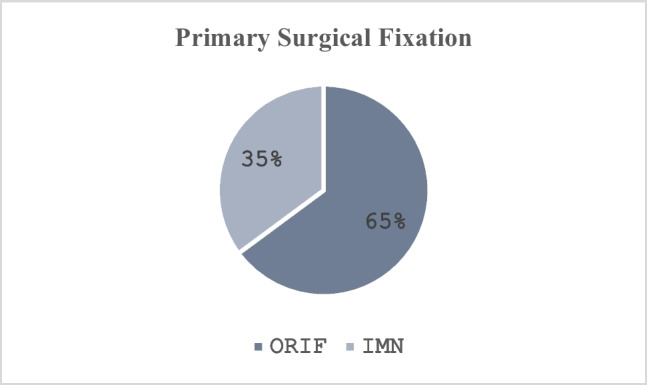
Delayed surgical fixation

**Fig. 8:**
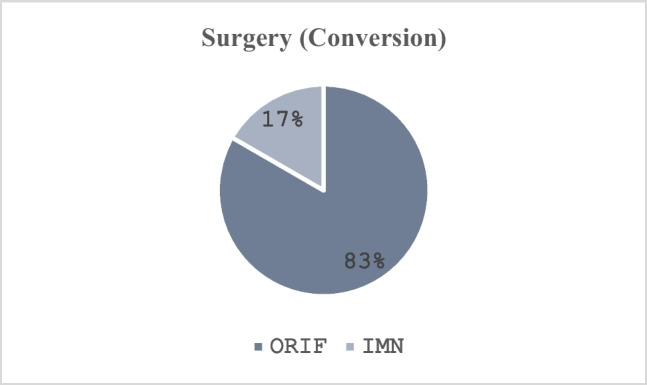
Delayed surgical fixation

Then, 37 patients underwent primary surgical fixation, of which there were 24 (64.9%) managed with open reduction and internal fixation (ORIF) and 13 (35.1%) treated with intramedullary nailing (IMN). For the 6 patients requiring delayed surgical fixation, there were 5 (83.4%) treated with ORIF and 1 (16.7%) treated with IMN (Fig. 7 and 8).

## Outcomes

Failure of conservative management occurred in 6/43 (13.95%) patients. The reasons for conversion to surgical intervention included further fracture displacement past acceptable tolerable levels, delayed union, and non-union. Univariate analysis of failed conservative treatment either leading to non-union or conversion to surgical fixation revealed associations with increased age, female gender, and increased AP translation of lateral radiographs. Successful non-operatively treated fractures showed a significant difference in AP translation on lateral radiographs with an average of 9.69 mm (IQR 4.90–14.05 mm) as compared to those that failed non-operative treatment with a mean of 22.61 mm (IQR 15.73–23.83 mm) (*p*-value 0.042). This parameter was considered an indication for primary surgical fixation with a mean of 20.02 mm (IQR 7.4–22.9 mm) for patients who underwent primary surgical fixation. It was noted that only a single fracture of the distal third of the humerus was managed non-operatively, whereas 13 distal third fractures were treated with primary surgical fixation (92.86%). This may indicate that distal third fractures inherently have a greater degree of initial displacement resulting in fracture characteristics that may be unsuitable for non-operative management.

## Discussion

The management of midshaft humerus fractures can often present a significant clinical challenge, particularly in determining the appropriate treatment approach, either to pursue primary surgical intervention or non-operative treatment. Multiple factors need to be considered when selecting the optimal treatment strategy. Conservative treatment is more prone to failure in cases where the fracture is a proximal oblique fracture, in patients over the age of 60 years and particularly amongst females [12]. Another consideration would be the initial displacement of the fracture. This study’s findings highlight the important role of initial radiographic parameters, specifically the anterior–posterior (AP) translation on lateral radiographs, as a predictor for possible failure of conservative treatment.

The results indicate a possible correlation between the degree of initial AP translation on lateral radiographs and the likelihood of non-operative treatment failure, necessitating subsequent surgical intervention. Patients who eventually required surgical intervention had significantly higher initial AP translation (mean 22.61 mm, IQR 15.73–23.83 mm) compared to those who were successfully treated non-operatively (mean 9.69 mm, IQR 4.90–14.05 mm). This marked difference suggests that fractures with greater initial displacement, specifically greater AP translation on the lateral radiograph, are less likely to achieve union with conservative methods alone. This may relate to the degree of interposition of soft tissue at the fracture site, such as may occur with penetration of one end of the fracture through the brachialis muscle. This study’s findings align with the notion that significant initial displacement may hinder the natural healing process [13]. In the context of humeral shaft fractures, non-operative management has traditionally been preferred due to its historically high union rates [14]. A significantly higher non-union rate has been confirmed in multiple studies than that identified by Sarmiento [4, 6, 7].

In patients suffering from non-union and the prolonged treatment duration that they need to endure, the effect on their quality of life and financial independence must be recognised. Additionally, the surgery for non-union is far more technically challenging and time consuming, demanding a disproportionate amount of theatre time and clinical resources [15]. Identifying patients at risk of conservative treatment failure may enhance patient satisfaction by enabling earlier definitive treatment. The ability to predict which fractures are unlikely to heal non-operatively allows for timely surgical intervention, potentially reducing the duration of pain, reducing disability, and improving patient outcomes.

Current literature on the management of humeral shaft fractures has primarily focused on patient demographics, fracture morphology, and comorbidities as predictors of treatment success or failure [7–9, 16, 17]. Our study seeks to contribute to this body of knowledge by demonstrating that a higher degree of AP translation on lateral radiographs may be associated with an increased rate of non-union and patients should be counselled regarding this when deciding on a treatment plan.

Our study’s findings support the premise that, whilst non-operative management is effective for many patients, those with significant initial displacement may derive greater benefit from primary surgical fixation. The clinical implications of this study offer valuable guidance on the treatment of humeral shaft fractures based on initial radiographic parameters. Given the elevated risk of treatment failure in patients with greater AP translation, clinicians should consider this radiographic finding when formulating treatment plans. This may relate to soft tissue interposition at the fracture site. Early appropriate surgical intervention would reduce the complications associated with delayed union, non-union, and the subsequent need for delayed surgical intervention.

Moreover, recognising increased AP translation as a predictive factor can aid in patient counseling. Patients with greater degrees of displacement can be informed about their increased risk of conservative treatment failure and the potential benefits of opting for surgical management upfront. This approach fosters shared decision-making, aligning patient expectations with clinical outcomes.

Kim et al. had suggested that certain radiographic parameters may be used to identify potential failure of non-operative management with the highest sensitivity and specificity were an AP angulation of greater than 11° and a M/L translation value of greater than 12 mm [10]. In our study, these were not realised in the patients who underwent conversion from non-operative treatment to surgical fixation. In our cohort, it was noted that there was an AP angulation with a mean of 5.25° (1.48–7.85°) and a M/L translation mean of 6.94 mm (2.45 mm, 7.73 mm). Although the radiographic parameters that they had mentioned were realised in our cohort of patients who underwent primary surgical fixation with an AP angulation mean of 11.53° (3.38–17°) and a M/L translation value mean of 19.67 mm (9.4–25.4 mm). This would support the notion of treating fractures with parameters greater than they had described with primary surgical fixation, due to the risk of failed non-operative treatment.

Despite its strengths, including the inter-observer reliability of radiographic analysis, this study has limitations. The retrospective design of the study may introduce selection bias, and the relatively small sample size limits the generalizability of the findings to the general population. Furthermore, whilst AP translation on lateral radiographs was a significant predictor of conservative treatment failure, other factors such as patient comorbidities, bone quality, and adherence to non-operative protocols were not evaluated as part of this study.

Future prospective studies with larger cohorts are needed to validate these findings and to investigate additional radiographic and clinical parameters that may predict treatment outcomes. Further research should aim to develop a comprehensive predictive model that integrates demographic, radiographic, and clinical factors to guide treatment decisions more effectively, to optimise successful fracture management.

## Conclusion

In conclusion, this study highlights the importance of initial radiographic parameters of displacement, particularly the AP translation on lateral radiographs in predicting possible failure of non-operative management for midshaft humerus fractures. Significant initial displacement is associated with a higher likelihood of requiring subsequent surgical intervention. These findings highlight the need for careful radiographic assessment at presentation and suggest that patients with substantial AP translation, of greater than 15 mm, may benefit from early appropriate surgical intervention to improve patient outcomes and prevent the prolongation of patient’s pain and disability.
